# A research parasite's perspective on establishing a baseline to avoid errors in secondary analyses

**DOI:** 10.1093/gigascience/giab015

**Published:** 2021-03-12

**Authors:** Ayush T Raman

**Affiliations:** Broad Institute of MIT and Harvard, 75 Ames St, Cambridge, MA 02142, USA; Department of Pathology and Center for Cancer Research, Massachusetts General Hospital and Harvard Medical School, Boston, MA 02114, USA

## Abstract

To enhance reproducibility in scientific research, more and more datasets are becoming publicly available so that researchers can perform secondary analyses to investigate questions the original scientists had not posited. This increases the return on investment for the NIH and other funding bodies. These datasets, however, are not perfect, and a better understanding of the assumptions that shaped them is required. The 2020 Junior Research Parasite Award recognized our work that showed that the signal-to-noise ratio in a particular dataset had not been investigated, leading to an erroneous conclusion in the original research. In this commentary, I share the process that led to the identification of the problem and hopefully provide useful lessons for other research parasites.

## Introduction

The Pacific Symposium on Biocomputing (PSB) Conference in 2020 recognized our contribution to rigorous secondary analysis with a Junior Parasite Award. This honor, initiated in 2017 and supported by *Gigascience* and the Gordon and Betty Moore Foundation, is awarded annually [1, 2] and is subsequently described in a published commentary that explains lessons learned while mining and re-analyzing the datasets. Below I summarize the steps that helped us.

My investigation into signal-to-noise ratios in a dataset began when I read an article that presented work to aid in understanding the molecular pathogenesis of Rett syndrome, a neurodevelopmental disorder caused by loss-of-function mutations in the *MECP2* gene. The MeCP2 protein is a methyl-binding epigenetic factor that controls the expression of several other genes. Previous microarray and RNA-seq studies have confirmed that thousands of genes are dysregulated in various brain regions in MeCP2-deficient mice. Thus it was surprising that the conclusion of this study was that the genes most likely to be misregulated by a mutated MeCP2 were genes longer than 100 kb [3]. This intriguing observation garnered much attention, but it was also biologically puzzling. Why would very long genes be more likely to be misregulated? Could the fact that neuronal genes tend to be longer than genes in other body tissues [4] have something to do with it?

My curiosity led me to re-analyze different MeCP2 datasets to reproduce the plots and results from the article. During this exercise, I realized that the previous studies did not use any statistical methods to establish a baseline against which the significance of the length-dependent changes could be measured—they assumed intra-sample variations to be equal to zero. This is not a good statistical practice. Just as in a wet lab where it is important to validate reagents prior to doing experiment to avoid having conclusions invalidated after discovering that one solution was bad, similarly, we need to check published datasets thoroughly. In the case of our study, this meant establishing the baseline variation. We found that when we established the baseline variation prior to analysis, the apparent bias toward misregulation of long genes by MeCP2 disappeared [5].

We then went a step further: we wondered about potential biases in other benchmark transcriptomic datasets, ones that did not include mutant MeCP2. We analyzed these using the authors' methods and found that these benchmark datasets also seemed prone to a “long gene bias.” This led us to hypothesize that the “long gene trend” might be an artifact of the microarray and RNA-seq technologies used to generate these datasets because both require PCR gene amplification. We therefore analyzed gene expression changes on the NanoString platform, which is not amplification-based. Here, we found that genes >100 kb were no more likely to be affected by MeCP2 mutations than genes of the shorter length. This supported our hypothesis that the previous observations of a long-gene bias resulted from amplification-based technologies and the failure to establish a proper baseline [5].

## Understand the Dataset and Statistical Methods Used by the Original Researchers

Downloading datasets from public repositories such as Gene Expression Omnibus is extremely easy, and it is tempting to take the data at face value and “simply” apply the algorithms that one is interested in. This temptation is all the stronger now because the widespread use of Google, Wikipedia, and similar platforms subtly persuades us that information is the same as knowledge. But it is not. To transform data into information, information into knowledge, and knowledge into understanding, we must approach the dataset with curiosity about its design, its purpose, the assumptions that guided its creation, and so forth [2]. This includes defining the dataset in terms of outcome variables (e.g., gene expression datasets), biological variables (e.g., genotype or phenotype information), and confounding covariates (e.g., data generated using different sequencing platforms or across different days). Other pertinent details include statistical methods and the assumptions that ground them, such as normality, heteroscedasticity, and independence of observations. These pieces of information establish a foundation for further analyses and enable us to ask better questions.

## Deconvolute Assumptions with Exploratory Data Analysis

**Figure 1:**
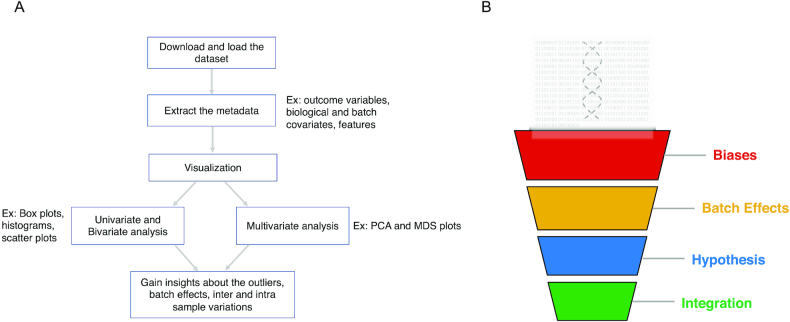
(A) Schematic of a subset of steps that are part of the exploratory data analysis workflow. Each box contains a description of the data analysis step and arrows indicate the progression through the analysis workflow. Ex: examples of respective data analysis step. (B) Schematic workflow of a typical secondary analysis project where big data is first analyzed to detect biases, batch effects in the dataset, gain insights and generate hypotheses, and integrate different datasets to get optimal results.

I recommend exploratory data analysis (EDA) to gain insight into the characteristics of the dataset. EDA uses different visualization techniques such as box plots, histograms, and scatter plots as a first step to learn whether the dataset is normalized, how the data points are distributed, and the total number of features in an assay. Next, I recommend using methods to reduce high-dimensional data, such as principal component analysis (PCA) and multi-dimensionality scaling (MDS). A well-labeled PCA plot has the power to reveal batch effects [6], labeling errors in the metadata sheet, inter- and intra-sample variations, and features that can distinguish groups within the sample (Fig. 1A).

During my efforts to understand the MeCP2 article, I wrote scripts that automate exploratory data analysis for all the datasets to investigate the plots methodically. This allowed our analysis to be consistent across different datasets of the same type. One of the eureka moments that came from such analyses was that the previous studies assumed that there was no intra-sample variation within the control group. This realization helped us develop a statistical approach to estimate intra-sample variation against which the significance of the length-dependent changes in mutant samples could be more accurately evaluated [5]. EDA revealed the degree of variability among different sample groups and informed further analyses required to investigate the background noise.

## Use Benchmark Datasets to Determine the Ground Truth

Because most scientific studies involve small sample sizes or low statistical power [7], a well-characterized benchmark dataset can be invaluable. In my project, it was by taking advantage of SEQC (Sequencing Quality Control) consortium datasets [8] that enabled us to see that the major source of technical variation was within samples. This led to our postulate that there might be PCR amplification bias.

In summary, any secondary data analysis project needs to methodically analyze possible biases and batch effects. Preliminary analysis will lead to hypotheses that should be tested using benchmark datasets. If required, publicly available genomic datasets should be used for data integration to elucidate biological pathways and mechanisms (Fig. 1B).

## Recognizing Error Is Part of Learning

In closing, I want to address the larger picture of this study and this award. “Parasite” does not have a positive connotation for most people, but the work of secondary analysis is an important part of expanding the body of knowledge. There have been many articles expressing concern over reproducibility in biomedical sciences [9], and I would argue that we can enhance scientific research by being more welcoming toward the discovery of mistakes and negative results. There is a bias towards positive new results [6], but if we do not publish negative results, mistakes can become embedded and lead to failed experiments and delay in scientific progress [10]. We are grateful to the researchers who generated and made the dataset accessible to the community, without which our research would not have been possible.

## Note from the Editors

The 2020 Research Parasite Award was held at the Pacific Symposium on Biocomputing on the Big Island of Hawaii, and in 2021 was presented at the virtual event via a livestream. The establishment of the award was a reaction to an editorial that presented arguments against data sharing, including that it promoted a system where “research parasites” (those who reuse datasets created by “frontline researchers”) would proliferate. As promoters of data sharing GigaScience Press has each year sponsored the Junior Parasite Award for postdoctoral, graduate, or undergraduate trainees and is again proud to support the award with travel grants and prize money. For more see the Research Parasite Awards website, https://researchparasite.com/.

## Data Availability

Not applicable.

## Abbreviations

*MECP2 or*MeCP2: Methyl-CpG Binding Protein 2; EDA: exploratory data analysis; kb: kilobase; MDS: multi-dimensionality scaling; NIH: National Institutes of Health; PCA: principal component analysis; PSB: Pacific Symposium on Biocomputing; SEQC: Sequencing Quality Control Consortium.

## Competing Interests

The author declares that he has no competing interests.
